# Experimental and Numerical Analyses of Stud Shear Connectors in Steel–SFRCC Composite Beams

**DOI:** 10.3390/ma15134665

**Published:** 2022-07-02

**Authors:** Kai Peng, Laijun Liu, Fangwen Wu, Ruizheng Wang, Song Lei, Xiaoyu Zhang

**Affiliations:** 1 School of Highway, Chang’an University, Xi’an 710064, China; 2018021013@chd.edu.cn (K.P.); liulj@chd.edu.cn (L.L.); 2018021016@chd.edu.cn (S.L.); xiaoyuzhang@chd.edu.cn (X.Z.); 2 Gansu Province Transportation Planning, Survey & Design Institute Co., Ltd., Lanzhou 730010, China; wrz1198656362@163.com; 3 China Railway First Survey and Design Institute Group Co., Ltd., Xi’an 710043, China

**Keywords:** steel fiber-reinforced cementitious composite (SFRCC), stud connector, shear performance, push-out test, FE analysis, load-slip curve, shear resistance

## Abstract

To investigate the shear performance and failure mechanism of stud shear connectors in steel fiber-reinforced cementitious composite (SFRCC) beams, six steel-SFRCC and six steel-normal strength concrete (NC) push-out specimens with two heights (80 mm, 120 mm) and three diameters (14 mm, 18 mm, 22 mm) of stud connectors were prepared. The experimental results revealed that the stud shearing failure was the main failure mode of all push-out specimens. In comparison to the steel-NC specimens, the development of cracks in the SFRCC beams was efficiently restrained due to the existence of high-strength steel fibers added to the normal concrete. The shear resistance and stiffness of studs in the steel-SFRCC beams were, respectively, 22.3% and 15.1% greater than those in the steel-NC specimens; however, their ductility was reduced, and the stud shear connectors failed in advance. The finite element (FE) model was developed and verified by push-out test results. FE analysis results indicated that the shear resistance of stud shear connectors was significantly improved with the increase in the concrete compressive strength, the stud diameter and tensile strength, whereas the aspect ratio of studs had a small impact on the ultimate resistance of stud shear connectors. Based on the as-obtained push-out experiment and FE analysis results, empirical formulas were presented to predict the load-slip curves and ultimate shear resistance of stud shear connectors in the steel-SFRCC specimens, and higher accuracy and a wider application range were obtained than with previous formulas.

## 1. Introduction

Steel-concrete composite structures combine the excellent material performance of steel and concrete and have the superiorities of high bearing capacities and a short construction period [1,2,3]. Connectors are used to resist the sliding and lifting effect between steel girders and concrete decks [4,5]. Stud connectors are the most common shear connectors in bridge engineering because of their same shear performance in all directions [6,7]. Cracks often appear in ordinary concrete decks of traditional composite structures, especially in the hogging moment area of continuous composite structures; thus, studs get corroded and seriously affect the bearing capacity and durability of composite structures [8,9]. Steel fiber-reinforced cementitious composite (SFRCC) has high strength, excellent tensile hardening ability, and good ductility [10,11,12,13]. Replacing ordinary concrete bridge decks with SFRCC bridge decks can easily solve the problem of cracking in traditional composite beam bridge decks, decrease the dead weight of composite beams, and increase the span of bridge structures.

Static tests and numerical simulations have been extensively adopted to probe the mechanical performance of studs embedded in normal strength concrete (NC). Buttry [14] performed a static experiment on steel-NC composite structures and demonstrated that the shear bearing resistance of studs in NC was mainly dominated by the material property of the concrete. Ding [15] studied the static behavior of studs through static tests and FE simulations and noted that the shear bearing capacity of each stud in the bidirectional specimens improved when the size and tensile strength of the studs increased, whereas the stud’s aspect ratio had no significant impact on the ultimate shear capacities of the studs. In addition, push-out tests have been widely performed [16]. It is suggested that the influence of steel beam type on the ultimate shear capacities of stud connectors cannot be ignored, and the quality of stud welding should be strictly controlled. Research on the mechanical behavior of stud connectors embedded in steel-NC composite beam is relatively mature, and some practical formulas for the ultimate shear resistance and load-slip curves of studs have been developed by considering stud geometry and concrete properties [17,18].

In recent years, steel fiber-reinforced cement-based composite materials have come to be used extensively in construction engineering. Researchers have proposed replacing NC with engineering cementitious composite (ECC) and ultra-high-performance concrete (UHPC) to enhance the longevity and traffic-carrying capacities of steel-concrete composite structure bridges [19,20,21]. Zhu [22] carried out two static tests on composite beams for different strengths of concrete and connectors and reported that ECC significantly limited the extension of cracks in the negative moment area of the composite structure and also enhanced the static performance of the composite structures. Guan [23] employed an FE model, which was verified by experimental results, to explore the mechanical behavior of studs in ECC and indicated that the interfacial bonding force between concrete decks and steel girders was conducive to improving the original shearing stiffness of ECC, and the diameter and tensile strength of studs effectively improved the stud shear capacity; however, opposite trends were noticed for the stud length and the ECC strength. Wang [24] experimentally investigated the mechanical properties of large-diameter studs embedded in UHPC and posited that UHPC was fitted properly with the large-diameter studs. An accurate formula was put forward by taking the stud diameter into account to calculate the load-slip relationships of studs in UHPC. In addition, the breaking of the composite structure mainly occurred at the boundary of studs and welds, and a simplified expression for the ultimate shearing resistance of studs in UHPC was proposed by taking the influence of the weld ring into account [25].

Although ECC and UHPC can improve the bearing resistance and service life of steel-concrete composite structures, they still have some deficiencies. ECC has good strain hardening capacity and ultra-high ductility; however, its compressive strength is almost similar to that of NC; thus, the ultimate shear capacities of steel-concrete composite beams cannot be significantly improved [26]. UHPC faces the problems of relatively high material preparation cost and complex structural design, and its low water-to-binder ratio causes large self-shrinkage [27]. Therefore, SFRCC could be a better choice for steel-concrete composite beams. As SFRCC has better crack resistance and toughness, the dominant crack may not be apparent until it is very close to the final fracture, and it can greatly improve the shear resistance of different components [28,29]. The steel matrix mainly transmits the load through the tensile strain hardening effect and the fibers bridging effect. The shear stress on the interface between the steel fibers and the surrounding matrix (bond strength between steel fibers and matrix) is a key parameter in the bridging effect, and concrete fails once the steel fibers either break or are completely pulled out from the concrete [30,31]. The presence of steel fibers supports the redistribution of tensile stress, prevents the propagation and opening of diagonal cracks, and the total yield bearing capacity of a steel fiber-reinforced composite beam can exceed the applied load when the steel bar starts to yield; thus, the composite beam exists in a multi-crack development mode and experiences an improvement in ductility [32,33]. SFRCC is mainly used for crack control and reinforcement works. However, the stress mechanism of steel-SFRCC composite beams is still unclear. Therefore, it is necessary to deeply explore the working performance and different influencing factors of studs embedded in steel-SFRCC composite beams to improve the promotion and application of steel-SFRCC composite structures.

Overall, the current research on the shear performance of stud connectors mainly focused on the studs in traditional steel-concrete composite structures, and it seems that there are few reports on the stud shear connectors in SFRCC composite beams. In order to investigate the shear performance and failure mechanism of stud shear connectors embedded in steel-SFRCC composite beams, the shear performances of six steel-NC and six steel-SFRCC specimens were investigated by push-out test for different stud diameters and lengths. The failure mode, load-slip relationship, shear capacities and stiffness of the push-out specimens and stud strains were examined. In addition, the FE models of different specimens were developed and verified by push-out test results, and the effects of concrete properties, stud diameter, stud yield strength, and stud aspect ratio on the shearing behavior of stud shear connectors were explored. Finally, according to the as-obtained push-out experiment and FE analysis results, empirical formulas were presented to forecast the load-slip curves and ultimate shear resistance of stud shear connectors embedded in the steel-SFRCC beams. This study presented a new idea for improving the mechanical performance of steel-concrete composite beams. In addition, the shear mechanism of studs in steel SFRCC composite beams was studied in detail. The research results could provide a reference for the popularization of SFRCC in composite beams.

## 2. Experimental Program

### 2.1. Specimens Design

**Figure 1:**
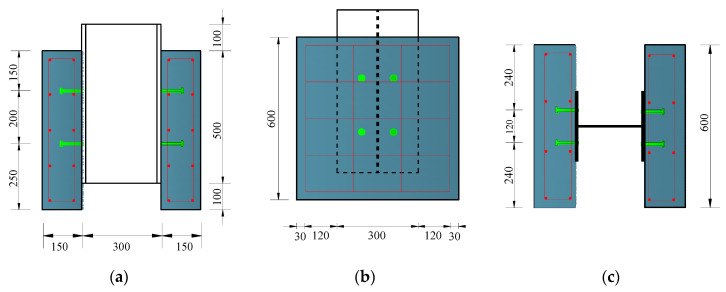
Geometry of push-out specimens: (**a**) front view; (**b**) side view; (**c**) plan view (unit: mm).

Six steel-NC and six steel-SFRCC push-out specimens were designed to explore the shear performance of stud shear connectors (Figure 1). The sizes of the concrete slabs were 600 mm × 600 mm × 150 mm, and an HRB400 reinforcement with a diameter of 8 mm was introduced into the concrete slabs as a stirrup. The dimension of the H-shaped steel beam made of Q345D bridge steel was 300 mm × 300 mm × 10 mm × 15 mm. Studs with different lengths (80 mm, 120 mm) and diameters (14 mm, 18 mm, 22 mm) were welded (vertical and horizontal spacings were 200 mm × 120 mm) to flange plates on each side of the H-shape steel beams. The specimen numbering format was “N/S-80/120-14/18/22”, where N represents NC, S represents SFRCC, 80/120 represents the stud length, and 14/18/22 represents the stud diameter. The bonding effect of concrete was not considered, and all specimens were coated with lubricating oil for the debonding treatment.

### 2.2. Specimen Fabrication

**Figure 2:**
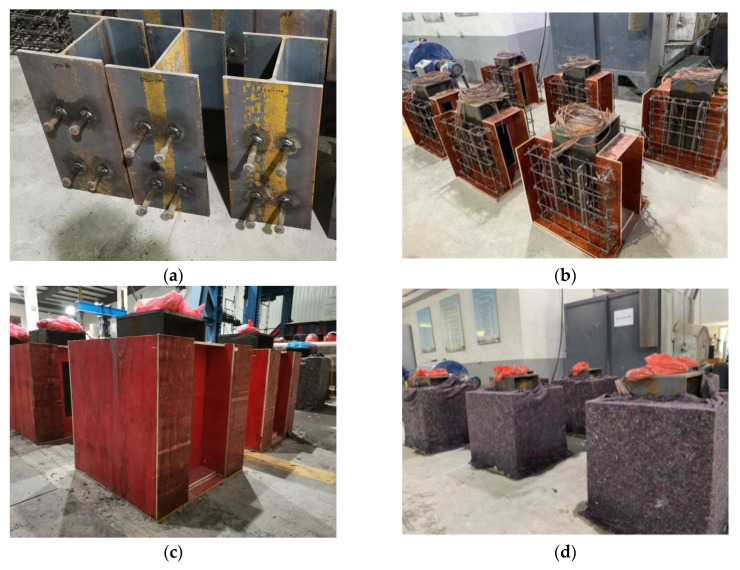
Specimen fabrication process: (**a**) stud welding; (**b**) mold assembly; (**c**) concrete pouring; (**d**) specimen maintenance.

The H-shaped steel beam was first cut into 600 mm segments to prepare test specimens, and the studs were welded by penetration welding at design locations on both sides of the steel plates. The stirrup cage was bounded to make the template according to the recommended design, the H-shaped steel beam and the stirrup cage were vertically assembled, and engine oil with a kinematic viscosity of 105 cSt was coated on the surfaces of the mold and the steel beams. Moreover, while pouring concrete, an immersion vibrator was used to ensure the homogeneous distribution of steel fibers within the concrete slabs. After the fabrication process, the specimens were covered with curing blankets and sprayed with water for 28 days under natural conditions. The preparation procedure of the experimental specimens is displayed in Figure 2.

### 2.3. Measurement of Material Properties

**Figure 3:**
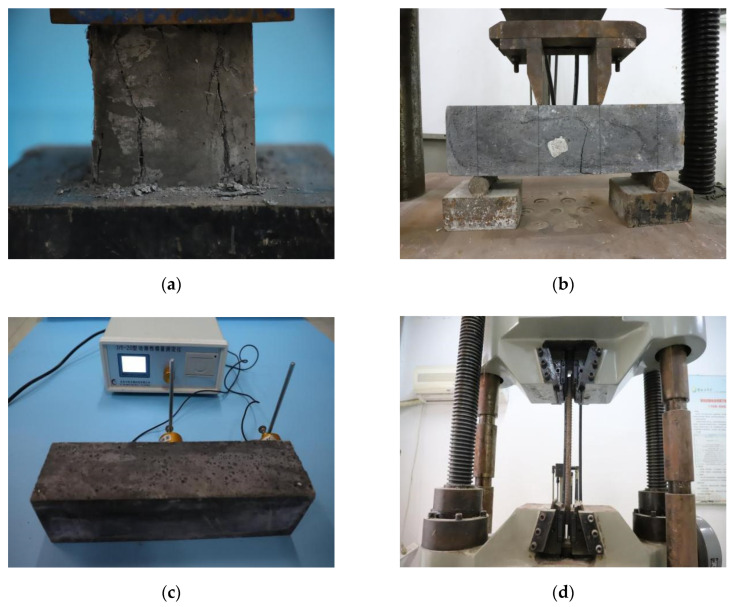
Measurement of mechanical properties: (**a**) concrete compression test; (**b**) concrete bending test; (**c**) concrete elastic modulus test; (**d**) rebar tensile test.

In the push-out test, the concrete was provided by Zhongde Xinya, a company in China. Table 1 presents the mixture proportions of NC and SFRCC. The main difference between NC and SFRCC was that there was silica fume and steel fiber in SFRCC, but not in NC. The hooked end steel fibers with a length of 30 mm and a diameter of 0.5 mm were mixed in the SFRCC. The steel fiber parameters are shown in Table 2. According to CECS 13:2009 [34], three groups of 100 × 100 × 100 mm^3^ cubic specimens and 150 × 150 × 550 mm^3^ prism specimens were fabricated to test the compressive and tensile strengths of concretes, and the elastic modulus of concretes was obtained by DT-20 Dynamic elastic modulus tester by resonance method (Figure 3). The material performance of NC and SFRCC are presented in Table 3. The mechanical properties of studs and steel beams were offered by the manufacturer, and the physical performance of each steel is listed in Table 4.

### 2.4. Test Setup

**Figure 4:**
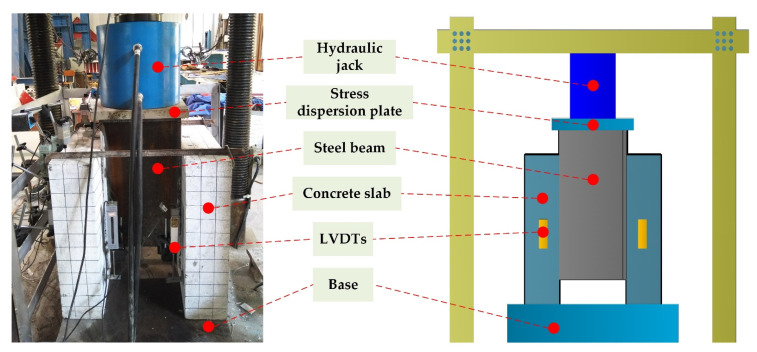
Push-out test setup.

The push-out test was carried out on a 500-ton press, and left-right symmetrical displacement gauges LVDTs were arranged at pre-selected positions of the concrete slabs and the H-shape steel beams to monitor the slippage between their interfaces. In order to ensure uniform stud stress during the test, the loading table was evenly paved with fine sand, and the stress dispersion plate was fixed on the undersurface of the hydraulic jack. The layout of the loading device and LVDTs are displayed in Figure 4. Preloading was carried out prior to the test. During formal loading, as the displacement increment was not detected until the relative slip reached 1 mm, loading was controlled by the applied load first, and the loading increment for each stage was 10 kN. The relative slip value corresponding to each loading stage was recorded. Plastic deformation expanded rapidly when the displacement was greater than 1 mm, and the data for every displacement increment of 0.1 mm were recorded until the specimen was destroyed.

## 3. Experimental Results

### 3.1. Failure Mode

**Figure 5:**
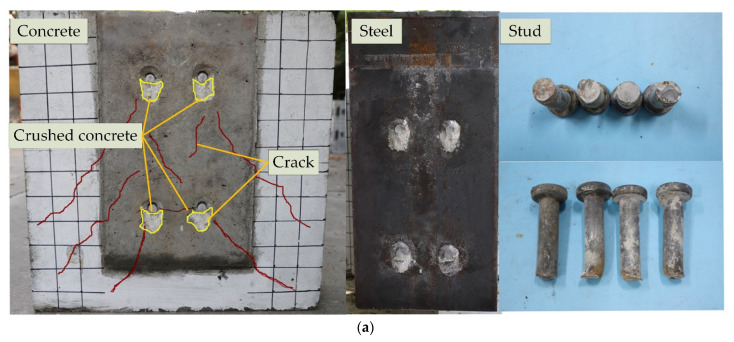

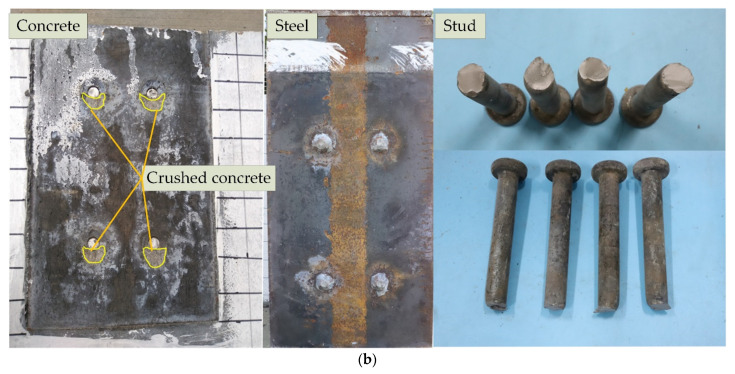
Failure modes of different test specimens: (**a**) N-80-18; (**b**) S-80-18.

The failure mode of the steel-concrete composite structures was mainly manifested as stud shearing failure and cracking of the concrete. When studs with smaller diameters and concrete with relatively higher compressive strength were used, the specimens ruptured due to the shearing failure of studs accompanied by a partial breaking of concrete slabs at the stud root. When studs with larger diameters and concrete with relatively lower compressive strength were used, the specimens were destroyed due to the cracking and breaking of concrete [35]. The failure modes of all steel-concrete composite beams are displayed in Figure 5. All 12 specimens experienced stud shearing failure with a smooth cross-section, and the concrete at the studs root also ruptured at the same time. The failure modes of the steel-NC and steel-SFRCC specimens were similar; however, oblique cracks in the NC slabs propagated further. There were no visible cracks in the SFRCC slab, and the local crushed area on the failure surface of the SFRCC slabs was smaller than that of the NC specimens, proving that the existence of steel fibers greatly limited the crack evolution in the SFRCC plates and the steel-SFRCC specimens took full advantage of the shearing performance of studs.

The shear carrying capacities of the steel-concrete composite structures were composed of two parts: (i) shear bearing capacity of studs and (ii) bearing capacity of concrete surrounding the stud root. During the static test, all specimens experienced a brittle failure. A long plateau period existed before the load reached the peak, and the studs were suddenly sheared with a loud noise. Subsequently, the load was greatly reduced, the concrete slabs were completely detached from the H-shape steel beams, and the push-out test was terminated.

### 3.2. Load-Slip Relationship

**Figure 6:**
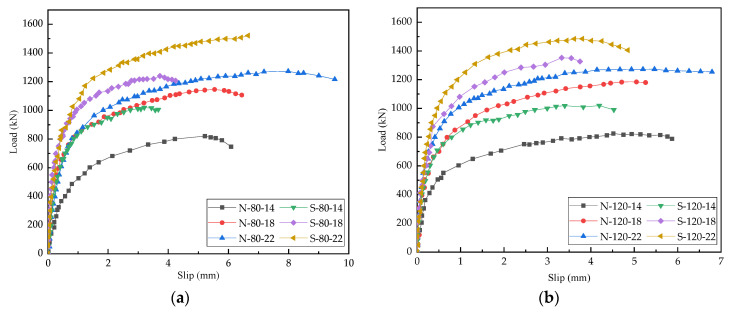
Load-slip curves of different stud lengths: (**a**) 80 mm; (**b**) 120 mm.

Figure 6a,b display the load-slip curves of different studs with heights of 80 mm and 120 mm, respectively. The curve consisted of an elastic phase, a plastic phase, and a failure phase. When the load did not exceed 46% of the shear bearing resistance, the load-slip curves showed a linear elastic relationship. As the load increased, the stud shear connectors entered the plastic phase, and the slip increased faster than the load. When the loading force was greater than the maximum bearing resistance, the specimen was damaged, and the load decreased sharply; however, the relative displacement continued to increase, and the load-slip curve in the failure stage became steeper and shorter.

As can be seen from Figure 6, the shear bearing resistance of studs embedded in the steel-concrete composite beams was significantly affected by the concrete compressive strength and the stud diameter. In the steel-NC specimens, the average shear bearing capacities of the studs with 14 mm, 18 mm, and 22 mm diameters were 822.54 kN, 1168.06 kN, and 1272.66 kN, respectively, and in the steel-SFRCC specimens, the corresponding values were 1021.15 kN, 1294.19 kN, and 1507.31 kN, respectively. The shear bearing capacities of studs embedded in the steel-SFRCC specimens increased by about 17.14% as compared to that in the steel-NC specimens; however, the average value of the maximum slip decreased by about 24.37%, indicating that the ductility of the steel-SFRCC specimens decreased and the stud connectors failed in advance. In the steel-SFRCC composite beams, the shear bearing capacities of the studs with 22 mm diameter was 47.62% and 16.47% higher than those of the studs with 14 mm and 18 mm diameters, respectively. Under the same concrete type and stud diameter, the ultimate shear capacities of the specimens with 80 mm and 120 mm stud lengths were the same. The studs transmitted the shearing force between steel beams and concrete decks through the root of stud connectors; thus, stud length had a small impact on the shear capacities of the steel-SFRCC composite beams. The shear resistance of each stud and the ultimate slippage of all specimens can be obtained from Figure 6, and the corresponding statistical data are presented in Table 5.

### 3.3. Shear Stiffness

Shear stiffness reflects the ability of studs to resist interfacial slip under stress, and it refers to the ratio of shear to relative slip. According to previous research, the secant slopes corresponding to the interfacial slippages of 0.2 mm and 2 mm were, respectively, used as the shear stiffness values of studs in the elastic and plastic phases [35,36,37]. The shear stiffness of all push-out specimens in the elastic and plastic are listed in Table 5. It is noticeable that the concrete type and the stud diameter have significant impacts on the stud shear stiffness. Taking the elastic shear stiffness as an example, the average shear stiffness of stud connectors embedded in the steel-SFRCC beams was 34.87% higher than that in the steel-NC composite beams. In the steel-SFRCC beams, the average shear stiffness of the stud connectors with 22 mm diameter was, respectively, 37.6% and 13.2% higher than those of the stud connectors with 14 mm and 18 mm diameters, and the average shear stiffness of the studs with 120 mm length was 17.2% higher than that of the studs with 80 mm length, indicating that stud length had a certain influence on the shear stiffness of studs.

### 3.4. Stud Strain

**Figure 7:**
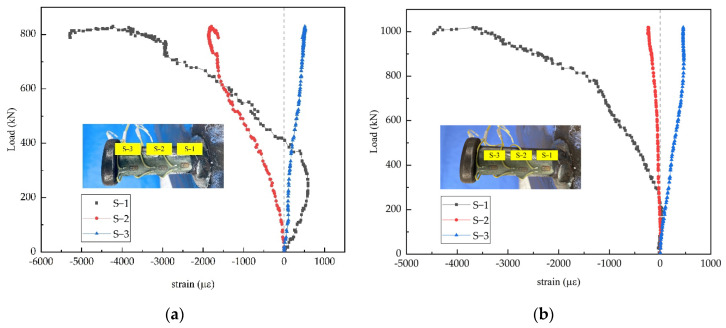
Load-strain relationship of studs: (**a**) N-120-22; (**b**) S-120-22.

The stud connectors experienced shear deformation and bending deformation simultaneously during the experimental process. As the flexural rigidity of the stud shear connectors was lower than their shear stiffness, the studs first produced bending deformation under a certain load; hence, the stud connectors were also subjected to the axial force [16,38]. Three strain gauges were uniformly arranged along the length of the stud to measure the stud strain, and the test data were automatically collected and recorded by the TDS-530 data collecting instrument during the test process. Figure 7 exhibits the load-strain curves along the lengths of the stud connectors, and the plotted load-strain curves had certain irregularities due to the complex state of the specimens. The studs had larger strains at the root and smaller strains at the end, indicating that the stud root was under a high-stress state and prone to fracture.

## 4. FE Analysis

### 4.1. FE Model

**Figure 8:**
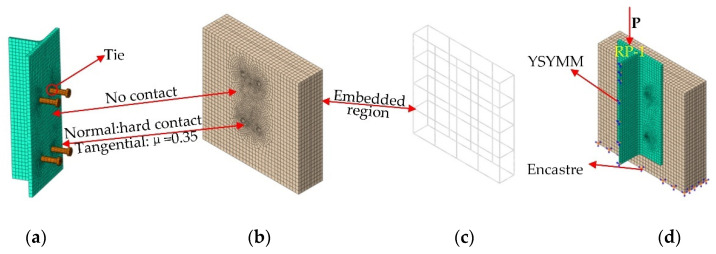
FE model: (**a**) steel beam and studs; (**b**) SFRCC slab; (**c**) rebar; (**d**) specimen.

The FE models of the push-out specimens were built by ABAQUS, and the standard solver was used to solve them. The half structures of the specimens were established considering the symmetry of their geometric dimensions and loading conditions. Concrete slabs, steel beams, and studs were simulated by the three-dimensional eight-node element (C3D8R), whereas the three-dimensional two-node truss element (T3D2) was applied to simulate stirrup cages, there were a total of 27,929 elements in the FE model. In order to ensure certain calculation accuracy and minimize the calculation cost, the structured meshing technique was adopted, and 2–5 mm meshes were used for the stud root and the concrete surrounding the studs, whereas 20 mm meshes were used for the remaining structure (Figure 8). The stress-strain curves suggested by Liao [30] was adopted for concrete, and the parameters of the concrete plastic damage model are presented in Table 6. The viscosity coefficient affected the simulation results to a certain extent. After trial calculations, the viscosity coefficient was selected as 0.0005. Steel has obvious elasticity and yield stage. When the stress reaches the yield point, the stress will not increase, but the strain can continue to increase. The ideal elastic-plastic model (double-line model) was employed for steel plates, studs, and steel bars (isotropic deformation occurred in a small range, and the Mises yield criterion was obeyed).

During the test, the bonding performance between the flange plates and the concrete slabs was not considered (the sides of the flange plate were smeared with lubricating oil); thus, the tangential and normal directions between the steel beams and the concrete slabs were set as a frictionless contact and a hard contact, respectively. The force between studs and concrete was divided into normal force and tangential bonding force. The normal direction was set as a hard contact to ensure the transmission of force, and a “penalty” friction formula with a factor of 0.5 was considered in the tangential direction to simulate the bonding force between studs and concrete. The reinforcement was embedded inside the concrete using the “embedded region” function of the ABAQUS interaction module. Symmetrical boundary conditions were created on corresponding symmetry planes to constrain the displacements perpendicular to the symmetry planes and the rotations of the symmetry planes. A “fully fixed” boundary condition (all degrees of freedom were restrained) was used at the bottom of the model. In order to facilitate load application and result extraction, a reference point RP1 was established at the top of the specimen, a coupling constraint was adopted between RP1 and the top surface of the model, and displacement loads were applied at RP1 according to a smoothed magnitude function.

### 4.2. Validation of FE Model

**Figure 9:**
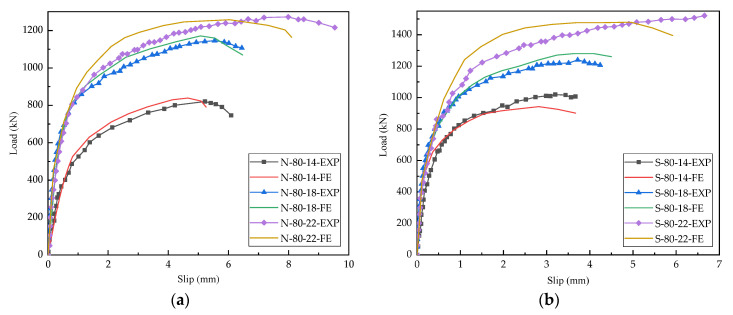
Load-slip curves of static test and FE simulations: (**a**) NC; (**b**) SFRCC.

The comparisons of the load-slip curves acquired from the static test and FE simulations are displayed in Figure 9. The maximum error of the stud shear capacity obtained from the FE and the push-out test was 2.8%. The curves calculated by the FE models were consistent with the trend of the test curves, especially in the initial loading stages. When the material entered the plastic stage, a certain deviation was noticed between FE and experimental values, and it might happen because the stress condition of the specimens was complex, and there were many influencing factors in this stage. However, in actual projects, the elastic proportional limit is generally taken as the design value of shear bearing resistance; thus, the deviation was within the acceptable range.

**Figure 10:**
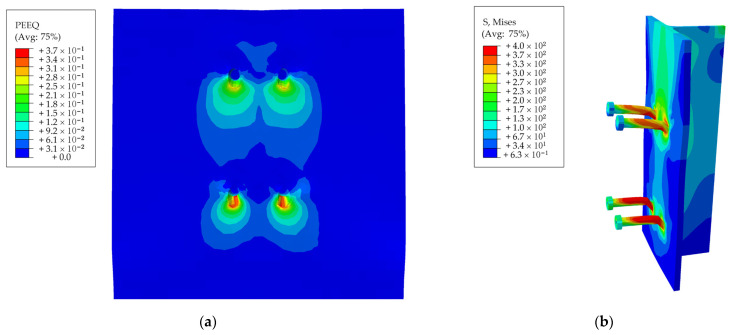
Results obtained from FE simulations: (**a**) concrete slab; (**b**) steel beam and stud.

Taking the S-80-22 specimen as an example, the stress nephogram of each component under ultimate load was shown in Figure 10. It can be seen from Figure 10 that under the ultimate load, the overall stress of the SFRCC slab was small, and the stress was concentrated near the root of the stud and gradually decreased radially towards the edge of the concrete slab. The stress on the upper part of the section steel flange plate was large, and there was large deformation in the horizontal direction, indicating that the upper part of the flange plate played a role in the horizontal restriction on the SFRCC slab. During the loading process, the concrete at the stud root was first locally crushed. With the increase in load, the stud got sheared and damaged; subsequently, the steel plate became warped and deformed. FE simulations could properly simulate the performance of the push-out test; thus, the obtained numerical results can be used for further research on the force transmission mechanism and parametric analysis of SFRCC stud connectors.

### 4.3. Influences of Different Parameters

For the purpose of revealing the shearing mechanism of stud connectors embedded in SFRCC, the above-discussed refined FE model was used for parametric analysis.

**Figure 11:**
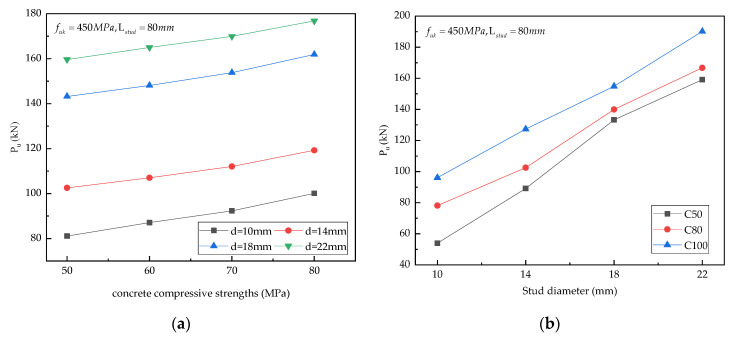

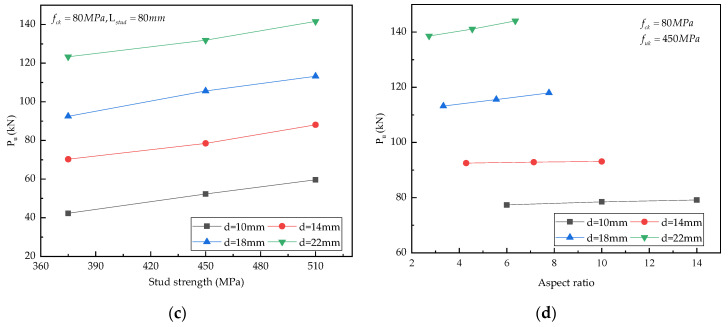
Parameter analysis: (**a**) concrete compressive strength; (**b**) stud diameter; (**c**) stud tensile strength; (**d**) stud aspect ratio. Note: d represents stud diameter; $L_{stud}$ represents stud length; $f_{\mathrm{ck}}$ represents concrete compressive strength; $f_{\mathrm{uk}}$ represents stud tensile strength.

(a) Concrete compressive strength: The shear bearing capacities of stud connectors for four different concrete strengths and four different stud diameters (10 mm, 14 mm, 18 mm, 22 mm) are presented in Figure 11a. It is noticeable that the ultimate shear capacities of studs were improved with the increasement of concrete compressive strength. The shear bearing capacities of stud connectors for the concrete strength of 80 MPa increased by 14.8%, 10.07%, and 5.71%, respectively, as compared to those for the concrete strength of 50 MPa, 60 MPa, and 70 MPa; however, the growth rate was not noticeably large.

(b) Stud diameter: The shear bearing capacities of studs for four different stud diameters and three different concrete strengths (50 MPa, 80 MPa, 100 MPa) are presented in Figure 11b. It is evident that the shear capacities of studs were enhanced significantly with the increase in stud diameter. The shear resistance of the stud connectors with 22 mm diameter was 126.19%, 61.71%, and 20.53% higher than those of the stud connectors with 10 mm, 14 mm, and 18 mm diameters, respectively, demonstrating that stud diameter played a crucial role in the shear capacities of studs.

(c) Stud tensile strength: The bearing capacities of stud connectors for the concrete compressive strength of 50 MPa, the stud diameters of 10 mm, 14 mm, 18 mm, 22 mm, and the ultimate stud tensile strength of 345 MPa, 420 MPa, and 550 MPa are presented in Figure 11c. The shear capacities of stud connectors with the tensile strength of 550 MPa were 12.15% and 9.29% higher than those of the stud connectors with the tensile strength of 345 MPa and 420 MPa, respectively, indicating that the shear bearing capacities of the studs enhanced with the improvement of their tensile strength; however, the increment was small.

(d) Stud aspect ratio: The bearing capacities of stud shear connectors with 12 different aspect ratios (length = 80 mm, 100 mm, 120 mm and diameter = 10 mm, 14 mm, 18 mm, 22 mm) for the concrete compressive strength of 50 MPa and the stud tensile strength of 550 MPa are presented in Figure 11d. When the stud diameter was constant, stud length had little impact on the shear bearing capacities of stud connectors, implying that the aspect ratio had no great influence on the shear bearing resistance of stud connectors within a certain range.

## 5. Theoretical Analysis

### 5.1. Load-Slip Curve

The load-slip curve of studs is a vital characteristic curve and reflects the studs’ mechanical performance change during the push-out test. The shear stiffness, ultimate shear resistance, and maximum slippage of studs could be acquired from their load-slip curves. Empirical curve formulas proposed by previous researchers are mainly applicable to ordinary concrete and high-graded concrete. There are few studies on the studs embedded in the SFRCC.

Ollgaard et al. [39]. conducted 48 two-slab push-out experiments to investigate the static performance of studs embedded in two types of concretes with different aggregates and expressed the load-slip curve for continuous loading as:

(1) $$\frac{P}{P_{u}}={\left(1-e^{-18\cdot S}\right)}^{0.4}$$

where *P* is the load on per stud and *S* is the relative slippage.

Xue et al. [16]. summarized the results of static tests to analyze the influences of different factors on the shearing performance of stud connectors and expressed the load-slip relationship of studs as:

(2) $$\frac{P}{P_{u}}=\frac{S}{0.5+0.97\cdot S}$$

Wang et al. [24]. performed static tests on two types of concrete to explore the influences of stud connectors size and concrete strength on the mechanical behavior of studs and put forward a practical load-slip equation by taking the stud diameter into account:

(3) $$\frac{P}{P_{u}}=\frac{S/d_{\mathrm{stud}}}{0.006+1.02\cdot S/d_{\mathrm{stud}}}$$

Zhang et al. [40]. carried out static tests and numerical simulations to explore the mechanical properties of stud connectors in steel fiber-reinforced concrete (SFRC) and drew a practical equation to forecast the load-slip curves of stud connectors:

(4) $$\frac{P}{P_{u}}={\left(1-e^{-0.2S}\right)}^{0.4}$$

Tong et al. [19]. tested six specimens of high-grade steel and UHPC under static loading. Considering the impacts of stud diameter and layout, a more accurate formula was presented to plot the load-slip curves of single-stud beams:

(5) $$\frac{P}{P_{u}}=\frac{S/d_{\mathrm{stud}}}{0.0092+0.93\cdot S/d_{\mathrm{stud}}}$$

It is noticeable that in the aforementioned research, the load-slip relationship of studs follows an exponential function. In the present work, considering the impact of concrete compressive strength, an exponential load-slip curve formula was proposed.

(6) $$\frac{P}{P_{u}}=0.95\times {\left(1-e^{-aS}\right)}^{b}$$

**Figure 12:**
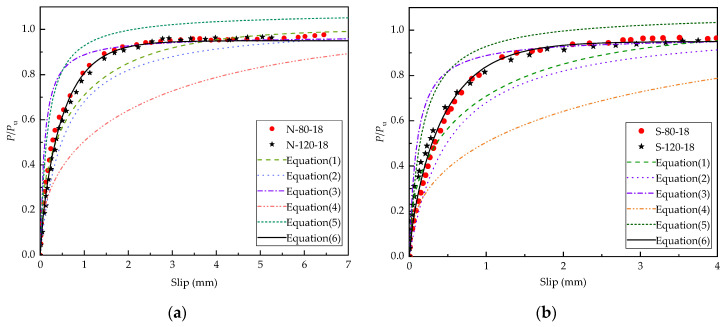
Comparison of push-out test and calculated load-slip curves: (**a**) NC; (**b**) SFRCC.

The average values of parameters *a* and *b* were obtained by fitting 12 groups of specimens (*a* = 1.63 for NC, *a* = 1.88 for SFRCC, and *b* = 0.75). The load-slip curve of the steel-NC and steel-SFRCC specimens with the studs diameter of 18 mm obtained from the push-out test and Equations (1)–(6) are displayed in Figure 12. It is obvious that the load-slip curve calculated by Equation (6) was closer to the test curve and has a wide range of applications.

### 5.2. Shear Resistance

The shear resistance is the most important index to measure the shearing performance of stud connectors. Some national codes provide relevant calculation formulas for the ultimate shear capacities of steel-concrete composite beams. In these codes, the shear resistance of studs is lower than the carrying capacities of concrete and studs, and this concept is different from the idea present in the current work. According to the push-out test and FE simulation results, a large gap was noticed between the ultimate shear capacities of stud connectors in SFRCC and NC under the same conditions, and no formula is currently available for evaluating the shear bearing resistance of studs embedded in SFRCC.

The Eurocode 4 [41] provides an expression for the calculation of studs’ shear resistance ($P_{u}$) embedded in steel-NC composite beams:

(7) $$P_{u}=0.29\alpha d^{2}\sqrt{f_{\mathrm{ck}}E_{c}}/\gamma_{v}\leq 0.8A_{s}f_{\mathrm{uk}}/\gamma_{v}$$

where $A_{s}$ represents the cross-sectional region of studs; $\gamma_{v}$ represents the security coefficient (1.25); α represents a parameter that depends on the length-diameter ratio ($\alpha =0.2(h_{s}/d+1)\leq 1$).

The American standard AASHTO LFRD [42] calculated the $P_{u}$ of single studs as:

(8) $$P_{u}=\varphi_{\mathrm{sc}}0.5A_{s}\sqrt{E_{c}f_{c}^{\prime }}\leq \varphi_{\mathrm{sc}}A_{s}f_{\mathrm{uk}}$$

where $f_{c}\prime$ represents the cylinder strength of concrete, and $\varphi_{\mathrm{sc}}$ represents the resistance reduction factor (0.85).

In China, the code GB50017-2017 [43] stipulates that $P_{u}$ can be defined as:

(9) $$P_{u}=0.43A_{s}\sqrt{E_{c}f_{\mathrm{ck}}}\leq 0.7A_{s}f_{\mathrm{uk}}$$

Ollgaard et al. [39] determined the shear capacities of studs based on the material properties of concrete:

(10) $$P_{u}=1.106A_{s}{f_{c}^{\prime }}^{0.3}E_{c}{}^{0.44}$$

Zhang et al. [40] conducted experiments and FE simulations to calculate the $P_{u}$ of studs embedded in SFRC and proposed a practical calculation formula for:

(11) $$P_{u}=\min\left(0.76A_{s}f_{u,\;}0.5A_{s}\sqrt{f_{c}E_{c}}\right)$$

Wang et al. [44] considered the influence of stud length on the shearing behavior of studs, and obtained the shear resistance calculation formula of studs based on a total of 80 domestic and foreign models:

(12) $$P_{u}=17.31A_{s}f_{u}{\left(\frac{h_{s}}{d_{s}}\right)}^{0.27}{\left(\frac{E_{c}}{E_{s}}\right)}^{1.75}{\left(\frac{f_{c}}{f_{u}}\right)}^{0.14}$$

In the abovementioned research, larger errors in calculation results could be attributed to two main factors: (i) The material value was conservative, and studs were calculated with their yield strength instead of their ultimate tensile strength. (ii) The calculation concept was conservative, and studs were considered when they got sheared and damaged. Moreover, the shear resistance of concrete was adjusted as a factor or not considered; however, in practical situations, concrete acts together with studs. Due to the existence of steel fibers, SFRCC has high compactness and few internal defects. The fibers bridging effect in SFRCC can restrict the development of cracks and increase the binding force and friction between the reinforcement and the matrix, causing an improvement in the matrix strength. Therefore, based on the push-out experiment and FE simulation results, a calculation expression of stud connectors’ shear resistance was put forward by considering the synergistic influence of concrete and studs.

(13) $$P_{u}=\left(0.85A_{s}f_{\mathrm{uk}}+1.25A_{s}f_{c}\right)/\lambda$$

where λ is the partial parameter of resistance (1.24).

**Figure 13:**
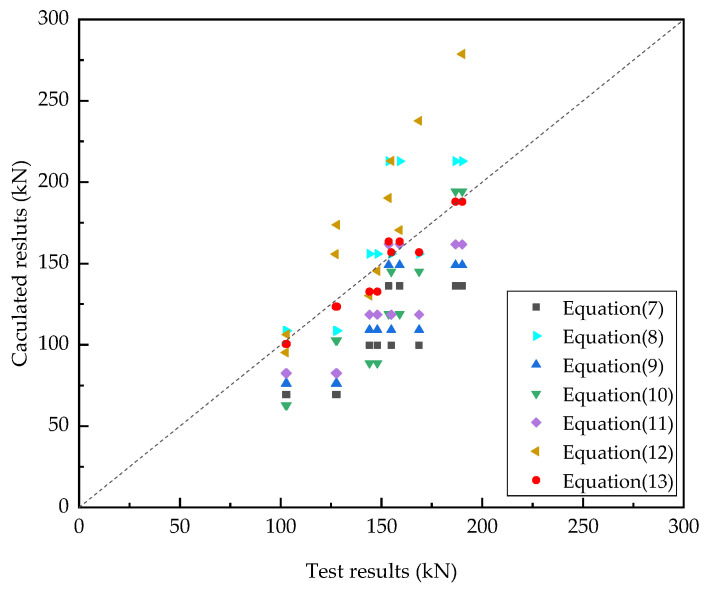
Comparison of the push-out test and calculated results of stud shear resistance.

Figure 13 displays a comparison of the static test and calculated results of stud shear resistance. The shear resistance of studs obtained by Wang et al.’s formula was found to be too large. The formula recommended by the AASHTO LFRD code was relatively accurate; however, the error fluctuated greatly. The formulas proposed by other researchers were conservative and underestimated the shear resistance of studs. It was found that Equation (13) had higher accuracy and wider applicability than the other formulas.

## 6. Conclusions

Push-out tests and FE simulations with different parameters were conducted to research the shear performance and failure mechanism of stud shear connectors embedded in steel-NC and steel-SFRCC composite beams. This study could promote the application of steel-SFRCC composite beams in civil engineering. The main findings of this research are listed below.

(1) All steel-NC and steel-SFRCC push-out specimens ruptured due to stud shearing failure with a smooth cross-section, and the concrete at the corresponding position of the stud root was also crushed at the same time. The failure modes of the steel-NC and steel-SFRCC push-out specimens were similar; however, oblique cracks propagated further in the NC slabs. Contrary to the steel-NC specimens, there were no obvious cracks in the SFRCC slabs.

(2) The mechanical performance of the stud shear connectors was mainly dominated by the concrete compressive strength and the stud diameter and tensile strength. The studs had larger strains at the root and smaller strains at the end, indicating that the stud root was under a high-stress state and prone to fracture.

(3) Based on the as-obtained push-out experiment and FE simulation results, empirical formulas to predict the load-slip curves and shear resistance of stud shear connectors in steel-SFRCC were presented. The stud connectors load-slip curve calculation formula considered the effect of the concrete strength factor, and the stud shear resistance calculation formula was suitable for both NC and SFRCC and had higher precision and a wider application range than previous formulas.

## Figures and Tables

**Table 1:** The mixture proportions of concretes.

Component	Mix Quantity (kg/m^3^)	
	NC	SFRCC
Cement P.O 52.5	453	669
Water	174	182
Broken stone 5~20 mm	1109	987
Sand 0~5 mm	597	524
Superplasticizer	4.23	6.98
Silica fume	-	80
Steel fiber (%)	-	148 (2%)

**Table 2:** Steel fiber parameters.

Fiber Type	L (mm)	D (mm)	L/D	Tensile Strength (MPa)	Elastic Modulus (GPa)	Density (kg/m^3^)
Hooked end steel fiber	16	0.2	80	2840	200	7800

Note: L = length of steel fiber; D = diameter of steel fiber.

**Table 3:** Material performance of concrete.

Concrete	Compressive Strength (MPa)	Tensile Strength (MPa)	Elastic Modulus (GPa)	Poisson’s Ratio
NC	51.2	1.83	36.5	0.2
SFRCC	115.4	17.6	45.3	0.2

**Table 4:** Mechanical performance of steel.

Material	Elastic Modulus (GPa)	Yield Strength (MPa)	Tensile Strength (MPa)	Poisson’s Ratio
Q345	206	349	468	0.3
HRB400	200	400	570	0.3
Studs of 16 mm	206	380	530	0.3
Studs of 19 mm	206	380	540	0.3
Studs of 22 mm	206	380	550	0.3

**Table 5:** Push-out test results.

Specimens	Pmax (kN)	Pu (kN)	Su (mm)	Load_0.2_ (kN)	k_0.2_ (kN/mm)	Load_2_ (kN)	k_2_ (kN/mm)
N-80-14	820.24	102.53	6.16	22.93	114.64	85.07	42.54
N-80-18	1152.48	144.06	6.45	56.67	283.36	122.39	61.20
N-80-22	1272.46	159.06	9.24	47.40	236.98	127.91	63.96
N-120-14	824.85	103.11	5.87	47.64	238.18	89.50	44.75
N-120-18	1183.63	147.95	5.26	65.52	327.59	127.67	63.84
N-120-22	1272.86	159.11	6.79	65.13	325.65	141.75	70.88
S-80-14	1018.66	127.33	3.67	53.55	267.74	118.68	59.34
S-80-18	1239.01	154.88	4.24	75.13	375.66	141.75	70.88
S-80-22	1520.93	190.12	6.66	72.45	362.26	160.41	80.21
S-120-14	1023.54	127.94	4.69	62.01	310.04	117.29	58.65
S-120-18	1349.36	168.67	3.75	75.42	377.09	156.31	78.16
S-120-22	1493.69	186.71	7.07	86.67	433.34	175.64	87.82

Note: Pmax is the maximum load, Pu is the ultimate shear resistance of each stud, Su is the maximum relative slippage, Load_0.2_ and Load_2_ are the loads for the slips at 0.2 mm and 2 mm, respectively, k_0.2_ and k_2_ are the shear stiffness values of studs for the slips at 0.2 mm and 2 mm, respectively.

**Table 6:** Plastic damage model parameters.

Expansion Angle	Eccentricity	$f_{b0}/f_{c0}$	K	Viscosity Coefficient
38	0.1	1.16	0.6667	0.0005

## Data Availability

Not applicable.

## References

[1] Nethercot D.A. (1996). Composite steel and concrete structural members: Fundamental behaviour. Eng. Struct..

[2] Lowe D.L., Roy K., Das R., Clifton C.G., Lim J.B.P. (2020). Full scale experiments on splitting behaviour of concrete slabs in steel concrete composite beams with shear stud connection. Structures.

[3] Nie J.G., Cai C.S. (2003). Steel–concrete composite beams considering shear slip effects. J. Struct. Eng..

[4] Pavlovic M., Markovic Z., Veljkovic M., Budevac D. (2013). Bolted shear connectors vs. headed studs behaviour in push-out tests. J. Constr. Steel Res..

[5] Han Q.H., Wang Y.H., Xu J., Xing Y. (2015). Static behavior of stud shear connectors in elastic concrete–steel composite beams. J. Constr. Steel Res..

[6] Qi J.N., Hu Y.Q., Wang J.Q., Li W.C. (2019). Behavior and strength of headed stud shear connectors in ultra-high performance concrete of composite bridges. Front. Struct. Civ. Eng..

[7] Wu F.W., Tang W.L. (2021). Experimental investigation on the static performance of stud connectors in steel-HSFRC composite beams. Materials.

[8] Tong L.W., Liu Y., Sun B., Chen Y.Y., Zhou F., Tian H., Sun X.D. (2014). Experimental investigation on mechanical behavior of steel-concrete composite beams under negative bending. J. Build. Struct..

[9] Fan J.S., Nie J.G. (2005). Effects of slips on load-carrying capacity of composite beams under negative bending. Eng. Mech..

[10] Song J.D., Duy L.N., Manathamsombat C., Kim D.J. (2015). Effect of fiber volume content on electromechanical behavior of strain-hardening steel-fiber-reinforced cementitious composites. J. Compos. Mater..

[11] Xu C., Su Q.T., Masuya H. (2017). Static and fatigue performance of stud shear connector in steel fiber reinforced concrete. Steel Compos. Struct..

[12] He Y.L., Wu X.D., Xiang Y.Q., Wang Y.H., Liu L.S., He Z.H. (2017). Mechanical behavior of stud shear connectors embedded in HFRC. Steel Compos. Struct..

[13] Fuseini M., Zaghloul M.M.Y., Elkady M.F., El-Shazly A.H. (2022). Evaluation of synthesized polyaniline nanofibres as corrosion protection film coating on copper substrate by electrophoretic deposition. J. Mater. Sci..

[14] Buttry K.E. (1965). Behaviour of Stud Shear Connectors in Lightweight and Normal-Weight Concrete. Ph.D. Thesis.

[15] Ding F.X., Yin G.A., Wang H.B., Wang L.P., Guo Q. (2017). Static behavior of stud connectors in bi-direction push-off tests. Thin-Wall. Struct..

[16] Xue W.C., Ding M., Wang H., Luo Z.W. (2010). Static behavior and theoretical model of stud shear connectors. J. Bridge Eng..

[17] Lee P., Shim C., Chang S. (2005). Static and fatigue behavior of large stud shear connectors for steel–concrete composite bridges. J. Constr. Steel Res..

[18] Shen M.H., Chung K.F. (2017). Structural behaviour of stud shear connections with solid and composite slabs under co-existing shear and tension forces. Structures.

[19] Tong L.W., Chen L.H., Wen M., Xu C. (2020). Static behavior of stud shear connectors in high-strength-steel–UHPC composite beams. Eng. Struct..

[20] Liu Y.M., Zhang Q.H., Bao Y., Bu Y.Z. (2019). Static and fatigue push-out tests of short headed shear studs embedded in engineered cementitious composites (ECC). Eng. Struct..

[21] Wang J.Y., Guo J.Y., Jia L.J., Chen S.M., Dong Y. (2017). Push-out tests of demountable headed stud shear connectors in steel-UHPC composite structures. Compos. Struct..

[22] Zhu L., Wang J.J., Li X., Tang L., Yu B.Y. (2020). Experimental and numerical study of curved SFRC and ECC composite beams with various connectors. Thin-Wall. Struct..

[23] Guan Y.H., Wu J.J., Sun R.J., Ge Z., Bi Y.F., Zhu D.Y. (2022). Shear behavior of short headed studs in steel-ECC composite structure. Eng. Struct..

[24] Wang J.Q., Qi J.N., Tong T., Xu Q.Z., Xiu H.L. (2019). Static behavior of large stud shear connectors in steel-UHPC composite structures. Eng. Struct..

[25] Huang Y., Chen S.M., Gu P. (2021). Static and fatigue behavior of shear stud connection embedded in UHPC. Structures.

[26] Hossain K.M.A., Alam S., Anwar M.S., Julkarnine K.M.Y. (2016). High performance composite slabs with profiled steel deck and engineered cementitious composite—Strength and shear bond characteristics. Constr. Build. Mater..

[27] Shao X.D., Deng L., Cao J.H. (2019). Innovative steel-UHPC composite bridge girders for long-span bridges. Front. Struct. Civ. Eng..

[28] Zaghloul M.M.Y., Mohamed Y.S., El-Gamal H. (2019). Fatigue and tensile behaviors of fiber-reinforced thermosetting composites embedded with nanoparticles. J. Compos. Mater..

[29] Zaghloul M.M.Y.M. (2018). Mechanical properties of linear low-density polyethylene fire-retarded with melamine polyphosphate. J. Appl. Polym. Sci..

[30] Liao W., Perceka W., Liu E. (2015). Compressive Stress-Strain Relationship of High Strength Steel Fiber Reinforced Concrete. J. Adv. Concr. Technol..

[31] Perceka W., Liao W., Wu Y. (2019). Shear Strength Prediction Equations and Experimental Study of High Strength Steel Fiber-Reinforced Concrete Beams with Different Shear Span-to-Depth Ratios. Appl. Sci..

[32] Wu K.R., Li S.J. (2006). Mechanical behaviors of hybrid steel fiber reinforced cementitious composites. J. Tongji Univ..

[33] Yang H.X., Li J., Huang Y.S. (2015). Study on the mechanical properties of high performance hybrid fiber reinforced cementitious composite (HFRCC) under impact loading. Key Eng. Mater..

[34] (2009). Standard Test Methods for Fiber Reinforced Concrete.

[35] Langarudi P.A., Ebrahimnejad M. (2020). Numerical study of the behavior of bolted shear connectors in composite slabs with steel deck. Structures.

[36] Ye H.W., Huang R., Tang S.Q., Zhou Y., Liu J.L. (2022). Determination of shear stiffness for headed-stud shear connectors using energy balance approach. Steel Compos. Struct..

[37] Qi J.N., Wang J.Q., Li M., Chen L.L. (2017). Shear capacity of stud shear connectors with initial damage: Experiment, fem model and theoretical formulation. Steel Compos. Struct..

[38] Huo J.S., Wang H.T., Li L., Liu Y.Z. (2019). Experimental study on impact behaviour of stud shear connectors in composite beams with profiled steel sheeting. J. Constr. Steel Res..

[39] Ollgaard J.G., Sluttter R.G., Fisher J.W. (1971). Shear strength of stud connectors in lightweight and normal-weight concrete. AISC Eng. J..

[40] Zhang Y.J., Liu A., Chen B.C., Zhang J.P., Pi Y., Bradford M.A. (2020). Experimental and numerical study of shear connection in composite beams of steel and steel-fibre reinforced concrete. Eng. Struct..

[41] (2004). Eurocode4: Design of Composite Steel and Concrete Structures.

[42] (2017). AASHTO LRFD Bridge Design Specifications, 8th ed.

[43] (2017). Standard for Design of Steel Structures.

[44] Wang J.F., Zhang A.P., Wang W.H. (2020). Effects of stud height on shear behavior of stud connectors. J. Zhejiang Univ..

